# Phenothiazinen-Dimesitylarylborane-Based Thermally Activated Delayed Fluorescence: High-Performance Non-doped OLEDs With Reduced Efficiency Roll-Off at High Luminescence

**DOI:** 10.3389/fchem.2019.00373

**Published:** 2019-05-29

**Authors:** Xiangyang Tang, Yanchun Tao, Hui Liu, Futong Liu, Xin He, Qiming Peng, Jinyu Li, Ping Lu

**Affiliations:** ^1^State Key Lab of Supramolecular Structure and Materials, Jilin University, Changchun, China; ^2^Key Laboratory of Flexible Electronics (KLOFE) and Institute of Advanced Materials (IAM), Jiangsu National Synergetic Innovation Center for Advanced Materials (SICAM), Nanjing Tech University (NanjingTech), Nanjing, China

**Keywords:** thermally activated delayed fluorescence, organic light-emitting diodes, non-doped device, high efficiency, low efficiency roll-off

## Abstract

We report a phenothiazinen-dimesitylarylborane thermally activated delayed fluorescence (TADF) molecule that exhibits high external quantum efficiency (EQE) in non-doped organic light-emitting diodes (OLEDs) at high luminescence. The non-doped device shows green electroluminescence with an emission peak of 540 nm and a maximum EQE of 19.66% obtained at a luminescence of ~170 cd m^−2^. The EQE is still as high as 17.31% at a high luminescence of 1,500 cd m^−2^ with small efficiency roll-off.

## Introduction

Thermally activated delayed fluorescence (TADF) materials are emerging as third-generation organic electroluminescence materials and are expected to replace Ir- or Pt-based phosphorescent complexes in practical application (Adachi, 2014; Reineke, 2014; Chen H.-W. et al., 2018). In organic light-emitting diodes (OLEDs), the singlet/triplet exciton branching ratio upon electrical excitation is generally believed to be 1:3 according to spin statistics. Phosphorescent materials can make the triplet state emissive *via* strong spin-orbit coupling caused by heavy metal atoms and are widely applied in full-color displays and white lightings (Zhang et al., 2015; Liu et al., 2016; Guo et al., 2017a; Miao et al., 2018a,b). However, the resource of Ir and Pt is limited. Therefore, developing low-cost alternatives is demanding. For fluorescent molecules, only the 25% singlet excitons can be emissive and the 75% triplet excitons are thus deactivated *via* thermal motion. Hence, how to utilize triplet exciton is the intrinsic issue in terms of boosting the efficiency of fluorescent OLEDs. TADF materials can up-convert the lowest triplet (T_1_) exciton to the lowest singlet (S_1_) excited state *via* reverse intersystem crossing (RISC) by absorbing thermal energy of surroundings provided that the energy splitting (Δ*E*_ST_) between T_1_ and S_1_ is sufficiently small, enabling the internal quantum efficiency (IQE) to approach unity without introducing noble metal elements (Endo et al., 2009; Uoyama et al., 2012; Zhang et al., 2012, 2014b). A general molecular design strategy toward small Δ*E*_ST_ proposed by Adachi et al. is linking electron donor and acceptor with high triplet energy in a twisted manner so that the highest occupied molecular orbital (HOMO) and the lowest unoccupied molecular orbital (LUMO) can be separated to get a small exchange energy (Endo et al., 2011; Lee et al., 2012). Though TADF materials can readily realize high device efficiency comparable to phosphorescent OLEDs, some critical issues still cannot be well resolved, which impede TADF materials toward practical application. One is that TADF materials normally need to be doped into appropriate host in device (Lee et al., 2012). The other is that the device efficiency decreases rapidly at high luminescence (Zhang et al., 2012, 2014a). The polarity, carrier transporting property, triplet energy of host, etc. can all remarkably influence TADF device performance (Lee et al., 2012; Komino et al., 2013; Li et al., 2016; Zhang J. et al., 2016). Hence, selecting or synthesizing proper host materials is necessary to obtain the desired device efficiency. Optimizing doping concentration is also needed in order to acquire high device performance (Lee et al., 2012; Zhang D. et al., 2016). A low doping concentration may result in incomplete energy transfer from host to dopant while a high doping level can cause concentration quenching. Both would lead to inferior device performance. Therefore, developing TADF materials that can achieve high efficiency at high luminescence in non-doped device is of great significance in terms of academic research as well as practical application. For TADF materials, the device efficiency roll-off at high luminescence and the necessity of fabricating doped device intrinsically inherit from two aspects: the fluorescence quenching in aggregate state and the triplet exciton quenching at high current density. A majority of TADF molecules are weakly emissive in solution while strongly emissive in aggregate states (Guo et al., 2017b,c). This is owing to the small overlap between HOMO and LUMO orbitals, which would result in a relatively slow fluorescence radiative decay constant that can barely compete with the active non-radiative processes such as molecular vibrations and motions in solution. In contrast, these non-radiative processes are greatly limited in aggregate state partially because of the rigid twisted molecular configuration. Consequently, the emission in aggregate state is greatly enhanced compared to that in solution. However, the D-A molecular structure of the TADF material would results in strong dipole–dipole interactions in the neat film and consequently induces fluorescence quenching. In addition, close π-π stacking can also quench fluorescence. Moreover, the weak binding energy of S_1_ exciton arising from the CT attribute of S_1_ state renders high probability of spin flip of the excited electron in singlet manifold, indicating remarkable intersystem crossing (ISC) from singlet to triplet. However, instead of being converted back to singlet state to produce fluorescence, the long-lived triplet exciton is subject to deactivation by intermolecular interactions, thermal motions, oxygen, etc., causing appreciable decrease of fluorescence efficiency (Méhes et al., 2012). Hence, the neat film of some TADF materials normally suffers from relatively low fluorescence quantum yield. As a result, TADF molecules usually need to be dispersed in proper host materials. On the other hand, the relatively slow RISC process from T_1_ to S_1_ induces triplet excitons to accumulate at high current density and go through various quenching processes such as triplet-singlet quenching and triplet-charge quenching. A fundamental solution for this is accelerating the RISC process from T_1_ to S_1_ to the extent of fluorescence decay rate, which seems impossible without introducing heavy atoms such as Ir or Pt. Even though reducing Δ*E*_ST_ is feasible to speed up the RISC process, a microsecond or even longer delay component still exists for TADF molecules (Uoyama et al., 2012; Zhang et al., 2014b). Herein, we propose an alternative strategy toward high-efficiency non-doped OLEDs with reduced efficiency roll-off at high luminescence. Introducing a steric hindrance group to increase intermolecular distance can reduce intermolecular interactions. Accordingly, fluorescence quenching as well as triplet exciton quenching caused by intermolecular interactions can be alleviated. In this work, we adopt dimesitylarylborane (Mes_2_B) as a steric repulsive electron acceptor. The vacant p orbital of boron atom endows it with electron-withdrawing ability. As a result, boron derivatives are widely applied as optoelectronic materials and sensors (Shirota et al., 2000; Yamaguchi et al., 2001; Jia et al., 2005; Welch et al., 2006; Bonn and Wenger, 2015; Hirai et al., 2015; Kitamoto et al., 2015, 2016a,b; Na et al., 2015; Numata et al., 2015; Suzuki et al., 2015; Dou et al., 2016; Hatakeyama et al., 2016; Park et al., 2016; Shiu et al., 2016; Chen et al., 2017; D'Aléo et al., 2017; Lee et al., 2017; Lien et al., 2017; Matsuo and Yasuda, 2017; Nakanotani et al., 2017; Neena et al., 2017; Chen D.-G. et al., 2018; Kim et al., 2018). The peripheral methyl groups around boron can shield the boron atom from the outer environment to protect it from protonic agents such as moisture. The additional effect of these methyl groups is their steric repulsion to adjacent molecules so that intermolecular interactions can be reduced. As a result, a high photoluminescence quantum yield (PLQY) in the neat film and reduced efficiency roll-off are expected. Using phenothiazine (PTZ) as electron donor, the Mes_2_B is attached to the *N* position of PTZ and the resulting molecule PTZMes_2_B presents a twisted configuration to ensure the separation between HOMO and LUMO. The PTZMes_2_B shows intense green emission with a relatively high PLQY of 65% in the neat film. We fabricated both non-doped and doped devices with doping level ranging from low to high concentration. The performance of doped device enhances as the doping concentration increases. The non-doped device exhibits higher efficiency than these doped devices with a maximum external quantum efficiency (EQE_max_) of 19.66%, which is acquired at a luminescence of ~170 cd m^−2^. The EQE can still remain 17.31% at a luminescence of ~1,500 cd m^−2^, showing only 12% efficiency roll-off. In contrast to the fact that a large majority of TADF materials only suit for doped device and suffer from severe efficiency roll-off, we have achieved high efficiency at high luminescence in the non-doped device and the device performance is comparable to some reported non-doped TADF OLEDs (Numata et al., 2015; Guo et al., 2017b,c; Yang et al., 2018; Rao et al., 2019).

## Experimental

### General Methods

All the reagents and solvents used for the synthesis and characterization were purchased from Aldrich and Acros and used without further purification. The ^1^H and ^13^C NMR data were recorded on a Bruker AVANCE 500 spectrometer at 500 and 125 MHz, respectively, using tetramethylsilane (TMS) as the internal standard and DMSO-D_6_ or CDCl_3_ as solvent. Elemental analysis was performed on a Flash EA 1112, CHNS-O elemental analysis instrument. The MALDI-TOF-MS mass spectra were measured using an AXIMA-CFRTM plus instrument. Thermal gravimetric analysis (TGA) was measured on a Perkin-Elmer thermal analysis system from 30 to 900°C at a heating rate of 10 K min^−1^ under a nitrogen flow rate of 80 ml min^−1^. Differential scanning calorimetry (DSC) was performed on a NETZSCH (DSC-204) unit from 50 to 380°C at a heating rate of 10 K min^−1^ under nitrogen atmosphere. UV–Vis spectra were recorded on a Shimadzu UV-3100 spectrophotometer. Steady-state photoluminescence spectra were measured by an RF-5301PC spectrophotometer. Time-resolved photoluminescence spectra were performed on Edinburgh spectrometer LP920 with 355-nm laser flash as excitation source. The PL lifetime was measured using an FLS920 spectrometer with a 375-nm picosecond pulsed light-emitting diode excitation source (pulse width: 898.3 ps). The PLQY (Φ) is measured by integrating sphere. Single crystal measurement was carried out at room temperature on a Rigaku RAXIS RAPID diffractometer equipped with a graphite monochromated Mo-Ka radiation source. The crystal structure was determined *via* SHELXL-97 software program.

### Electrochemical Measurement

The electrochemical properties (oxidation and reduction potentials) were carried out *via* cyclic voltammetry (CV) measurements by using a standard one-compartment, three-electrode electrochemical cell given by a BAS 100B/W electrochemical analyzer. Tetrabutylammoniumhexafluorophosphate (TBAPF6) in anhydrous dimethyl formamide (DMF) or anhydrous dichloromethane (0.1 M) were used as the electrolyte for negative or positive scan. A glass-carbon disk electrode was used as the working electrode, a Pt wire was used as the counter electrode, and Ag/Ag^+^ was used as the reference electrode together with ferrocene as the internal standard at a scan rate of 100 mV s^−1^. According to the literature, the formal potential of Fc^+^/Fc is 4.8 eV below vacuum (Pomrnerehne et al., 1995). All potentials relative to the Ag/Ag^+^ electrode obtained from CV measurement are eventually referenced against Fc^+^/Fc to calculate HOMO/LUMO energy levels. The HOMO/LUMO levels are calculated according to the following formalism:

(1) $$\begin{array}{r} E_{H\text{OMO}}=-\left(E_{\text{OX}}\text{ vs}.\text{Fc}/\text{F}{\text{c}}^{+}+4.8\right)\text{eV} \end{array}$$

(2) $$\begin{array}{r} E_{\text{LUMO}}=-\left(E_{\text{red}}\text{ vs}.\text{Fc}/\text{F}{\text{c}}^{+}+4.8\right)\text{eV} \end{array}$$

where the *E*_ox_ vs. Fc/Fc^+^ and *E*_red_ vs. Fc/Fc^+^ are oxidation and reduction onset potentials relative to Fc/Fc^+^ internal reference, respectively.

### Theoretical Calculations

The ground-state (S_0_) and the lowest singlet excited state (S_1_) geometries were optimized at the B3LYP/6-31G(d, p) level. Natural transition orbitals (NTOs) of both S_0_ → S_1_ and S_0_ → *T*_1_ were calculated using the TD-M06-2X/6-31G(d, p) method on the basis of the optimized ground-state configuration.

### Device Fabrication

ITO-coated glass was used as the substrate and the sheet resistance was 20 Ω square^−1^. The ITO glass substrates were cleaned with isopropyl alcohol, acetone, toluene, and deionized water; dried in an oven at 120°C; treated with UV-zone for 20 min; and finally transferred to a vacuum deposition system with a base pressure lower than 5 × 10^−6^ mbar for organic and metal deposition. The MoO_3_ was deposited at a rate of 0.1 Å s^−1^. The deposition rate of all organic layers was 1.0 Å s^−1^. The cathode LiF (1 nm) was deposited at a rate of 0.1 Å s^−1^ and then the capping Al metal layer (120 nm) was deposited at a rate of 4.0 Å s^−1^. A mask with an array of 2 × 2 cm openings was used to define the cathode. The electroluminescent (EL) characteristics were measured using a Keithley 2400 programmable electrometer and a PR-650 Spectroscan spectrometer under ambient condition at room temperature.

### Synthesis

#### Mes_2_BBr

Mes_2_BBr is synthesized according to literature (Na et al., 2015). ^1^H NMR (500 MHz, DMSO-D_6_, 25°C, TMS) δ (ppm): 7.51 (d, *J* = 8.2 *Hz*, 2H; Ar H), 7.39 (d, *J* = 8.2 *Hz*, 2H; Ar H), 6.85 (s, 4H; Ar H), 2.33 (s, 6H; CH_3_), 2.01 (s, 12H; CH_3_).

#### PTZMes_2_B

In a 100-ml round flask, a mixture of 10*H*-PTZ (995 mg, 5 mmol), Mes_2_BBr (2.02 g, 5 mmol), Na^t^OBu (960 mg, 10 mmol), PH^t^Bu_3_BF_4_ (72 mg, 0.25 mmol), and Pd_2_(dba)_3_ (137 mg, 0.15 mmol) was dissolved in toluene (30 ml) and stirred at 110°C for 24 h under N_2_. The reaction was quenched by water and extracted with dichloromethane. The organic layer was collected and evaporated. The residue was purified *via* column chromatography by using petroleum ether/dichloromethane (5:1, v/v) as eluent to afford bluish-green solid (3.35 g, yield: 90%). ^1^H NMR (500 MHz, DMSO-D_6_, 25°C, TMS) δ (ppm): 7.45 (d, *J* = 8.4 *Hz*, 2H; Ar H), 7.33 (d, *J* = 6.4 *Hz*, 2H; Ar H), 7.23 (d, *J* = 8.4 *Hz*, 2H; Ar H), 7.18 (t, *J* = 7.1 *Hz*, 2H; Ar H), 7.08 (t, *J* = 7.1 *Hz*, 2H; Ar H), 6.84 (d, *J* = 2.6 *Hz*, 2H; Ar H), 6.83 (s, 4H; Ar H), 2.26 (s, 6H; CH_3_), 1.99 (s, 12H; CH_3_); ^13^C NMR (125 MHz, CDCl_3_, 25°C, TMS) δ (ppm): 145.92 (C), 142.98 (C), 142.75 (C), 141.71 (C), 140.79 (C), 138.82 (CH), 138.64 (C), 128.26 (CH), 127.60 (CH), 126.95 (CH), 125.75 (C), 123.92 (CH), 120.28 (CH), 23.56 (CH_3_), 21.26 (CH_3_); MALDI-TOF MS (mass *m/z*): calcd for C_36_H_34_BNS, 523.25; found, 523.84 [*M*^+^]. Anal. calcd (%) for C_36_H_34_BNS: C, 82.59; H, 6.55; N, 2.68. Found: C, 82.57; H, 6.56; N, 2.67.

## Results and Discussion

### Synthesis

**Scheme 1:**
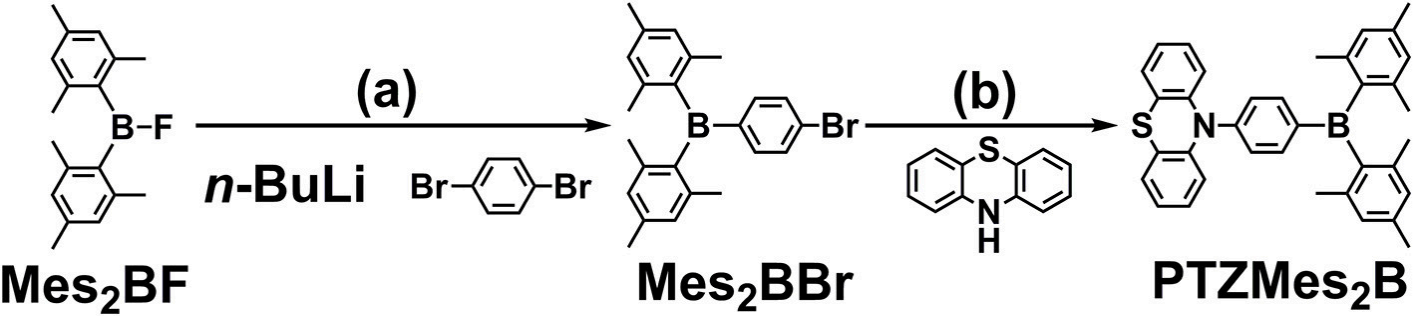
Synthesis of PTZMes_2_B: **(a)** 1,4-dibromobenzene, *n*-BuLi, −78°C, 3 h; Mes_2_BF in THF was added dropwise, stirring at room temperature overnight; **(b)** PTZ, Mes_2_BBr, Na^t^OBu, PH^t^Bu_3_BF_4_, Pd_2_(dba)_3_, toluene, 110°C, 24 h, under N_2_.

The synthesis of PTZMes_2_B is shown in Scheme 1. Mes_2_BBr is prepared the same way with literature (Na et al., 2015). Then PTZMes_2_B is readily acquired *via* Pd-catalyzed Ullmann coupling in high yield starting with PTZ and Mes_2_BBr as reactants. The target compound PTZMes_2_B is purified by column chromatography and sublimation. The chemical structure and purity are well verified *via* NMR, MS, elemental analysis, and crystallography.

### Thermal Properties and Morphology

Thermal property is investigated *via* thermogravimetric analysis (TGA) and DSC. As shown in Figure S1a, the decomposition temperature (*T*_d_, corresponding to 5% weight loss) of PTZMes_2_B is measured to be 381°C. The DSC curve of as-synthesized amorphous powder is displayed in Figure S1b. The weak endothermic signal at 84°C can be assigned to glass transition temperature (*T*_g_). The sharp exothermic peak at 157°C is attributed to crystallization (*T*_c_) and the intense endothermic peak at 262°C is the melting point (*T*_m_). To explore whether PTZMes_2_B can be applied in the non-doped device, film-forming ability is assessed by atomic force microscopy (AFM). Non-doped film of ~70 nm was prepared on quartz substrate by vacuum evaporation. An image of 5 × 5 μm area scanned by AFM is presented in Figure S2. Though the uniformity of film surface is inferior to that of some solution-processed oligomers (Yao et al., 2012), a root-mean-square (RMS) roughness of only about 0.72 nm is good enough for evaporation-deposited film. Therefore, PTZMes_2_B is suitable for non-doped device application.

### Electrochemical Properties and Single Carrier Device

**Figure 1:**
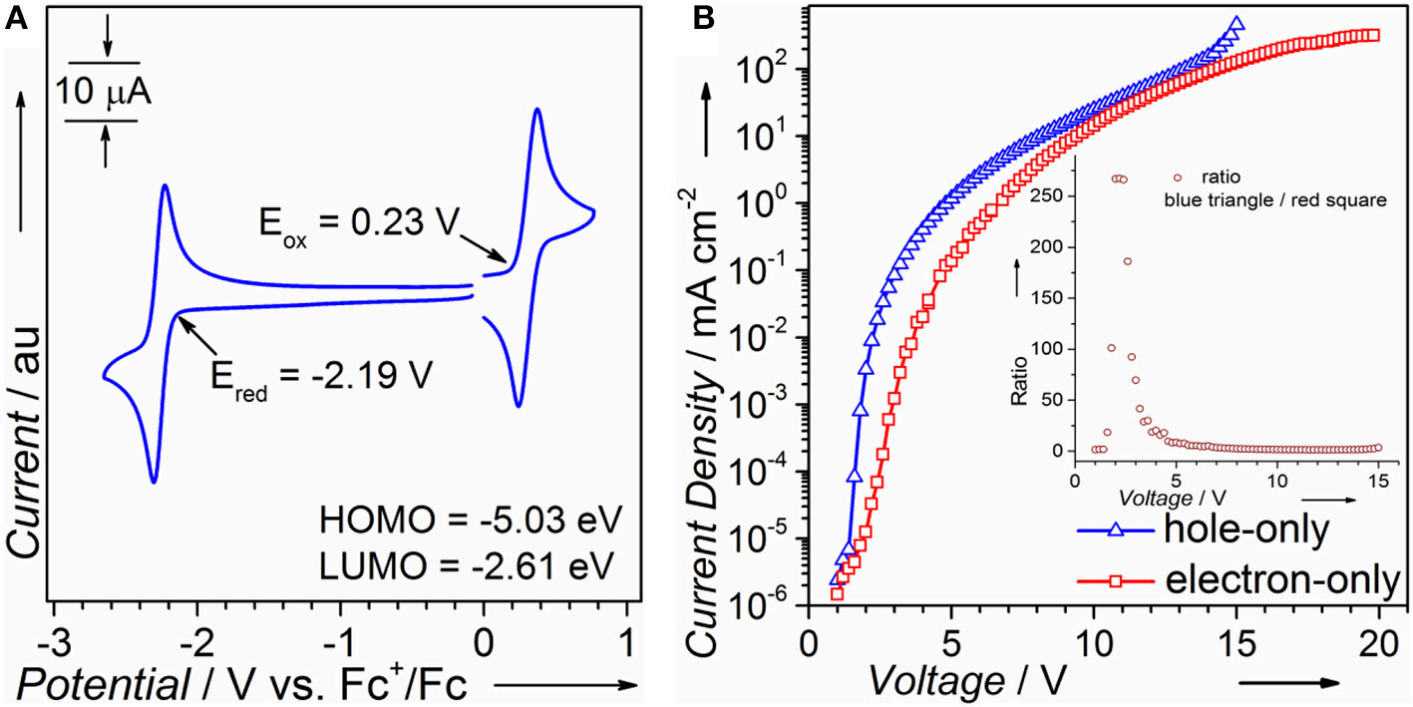
**(A)** Cyclic voltammetry and **(B)** single carrier device of PTZMes_2_B.

CV is performed to calculate HOMO/LUMO energy levels. As can be seen in Figure 1A, the oxidation and reduction onsets of PTZMes_2_B against ferrocenium/ferrocene (Fc^+^/Fc) redox couple are 0.23 and −2.19 V, respectively, corresponding to a HOMO energy of −5.03 eV and a LUMO energy of −2.61 eV (Equations 1 and 2). The coexistence of donor PTZ and acceptor Mes_2_B in one molecule yields proper HOMO/LUMO energy alignment that is in favor of both hole and electron injection from adjacent carrier transporting layers. We also fabricated single carrier devices to find out if PTZMes_2_B possesses balanced carrier transporting ability. The device structure of hole- and electron-only devices are ITO/HATCN (6 nm)/NPB (5 nm)/PTZMes_2_B (100 nm)/NPB (20 nm)/Al (100 nm) and ITO/TPBi (20 nm)/PTZMes_2_B (100 nm)/TPBi (5 nm)/LiF (1 nm)/Al (100 nm), respectively, where ITO is indium-tin oxide, HATCN is hexaazatriphenylenehexacarbonitrile, NPB is *N*,*N*'-bis(naphthalen-1-yl)-*N*,*N*'-bis(phenyl)-benzidine, and TPBi is 1,3,5-tris(1-phenyl-1*H*-benzimidazol-2-yl)benzene. Since we only aim to evaluate the carrier transporting property of PTZMes_2_B, but not carrier injection ability between electrodes and PTZMes_2_B, we have to eliminate energy barriers between electrodes and PTZMes_2_B. Hence, for the hole-only device, a thin layer of HATCN (6 nm)/NPB (5 nm) is inserted between the anode ITO and PTZMes_2_B layer to facilitate hole injection. The NPB (20 nm) layer between PTZMes_2_B and cathode Al is used to block electrons. Similarly, for the electron-only device, a thin layer of TPBi (5 nm)/LiF (1 nm) inserted between PTZMes_2_B and cathode Al is applied to enhance electron injection and the TPBi (20 nm) layer between PTZMes_2_B and the anode ITO is designed to prevent hole injection. For both hole- and electron-only devices, the PTZMes_2_B layer is much thicker than the NPB or TPBi layers so that the carrier mobility of the whole device is predominated by PTZMes_2_B. The result is shown in Figure 1B; the inset shows the value derived from current density of the hole-only device divided by that of the electron-only device. At any given voltages within the range of our measurement, we can see in Figure 1B that the current density of the hole-only device is always higher than that of the electron-only device, indicating that hole mobility of PTZMes_2_B is better than electron mobility. The difference between hole and electron mobility is quite large at low voltages such as 3 V at which the current density of the hole-only device is ~70 times that of the electron-only device. However, with increasing voltages, such difference gradually decreases, as can be seen in the inset of Figure 1B. For example, at a voltage of 4 V, the ratio (current density of the hole-only device over that of the electron-only device) is only ~20; at a voltage of 7 V, current density of the hole-only device is only ~1.5 times higher than that of the electron-only device. Nevertheless, PTZMes_2_B displays relatively balanced carrier mobility. Such bipolar nature of the D-A molecule is beneficial for balanced charge injection and recombination in the emitting layer, which can alleviate exciton quenching by the free charges. In addition, the bipolar character can widen the carrier recombination zone in the emissive layer, which may reduce the adverse effect of concentration-induced exciton quenching.

### Theoretical Calculations

**Figure 2:**
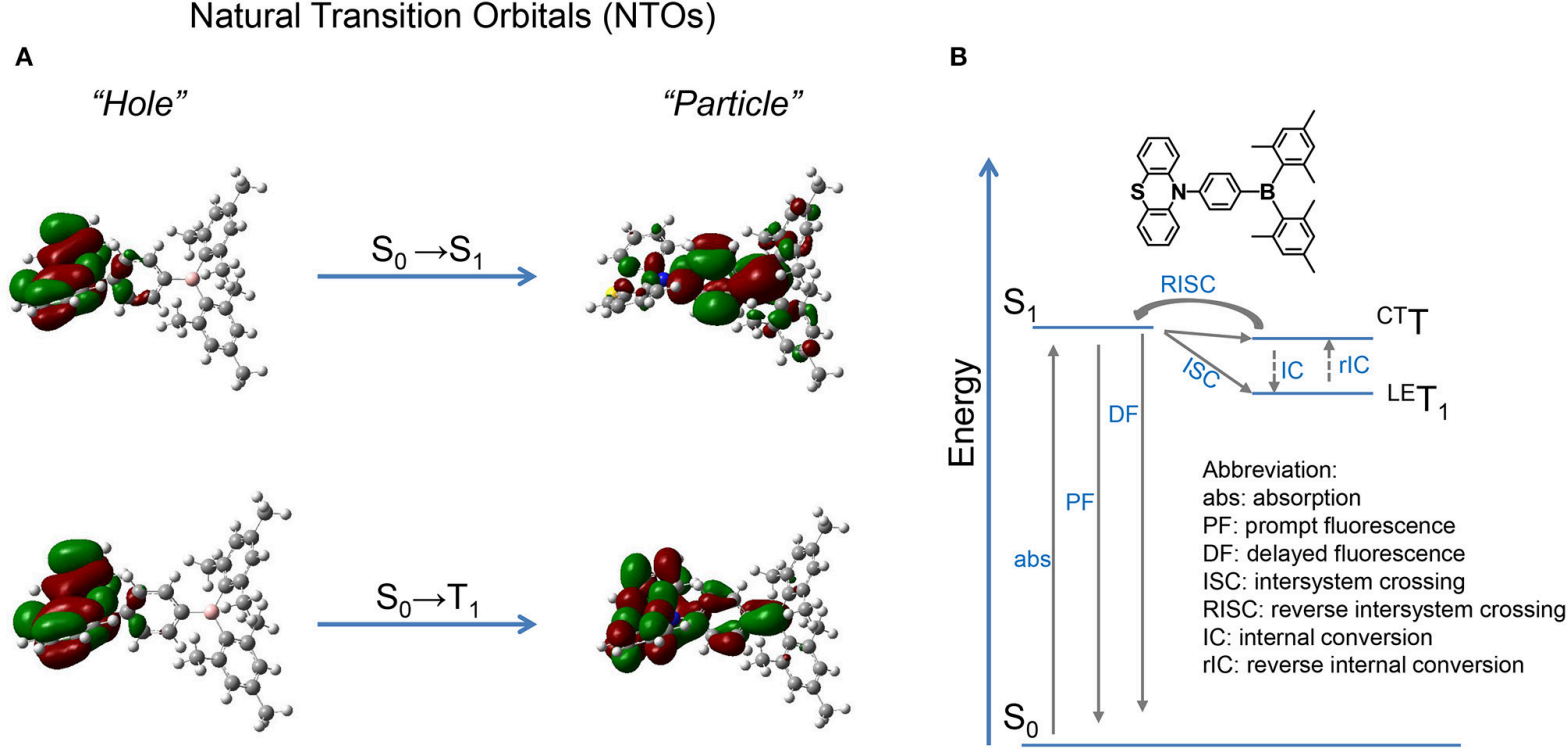
**(A)** NTOs of PTZMes_2_B; **(B)** possible TADF route.

NTOs are calculated by the TD-M062X/6-31G(d, p) method based on the optimized configuration of ground state. As shown in Figure 2, for the S_0_ → S_1_ transition, the hole and particle distribute separately on the donor PTZ and acceptor dimesitylboryl, respectively, revealing the CT attribute of S_1_ state. On the other hand, the hole and particle of S_0_ → *T*_1_ overlap on the donor PTZ moiety, disclosing the locally excited (LE) property of T_1_ state. The calculated S_1_ and T_1_ energies are 3.57 and 3.41 eV, respectively, resulting in a Δ*E*_ST_ of 0.16 eV. The binding energy of the LE exciton is generally large, which is adverse for spin flip of the excited electron, while the binding energy of the CT exciton tends to be small, which is beneficial for spin flip. Therefore, a reasonable explanation of the TADF emission is that the ^3^LE exciton is firstly up-converted to ^3^CT excited state and then goes through RISC process to the S_1_ state followed by delayed fluorescence.

### Crystal Structure

**Figure 3:**
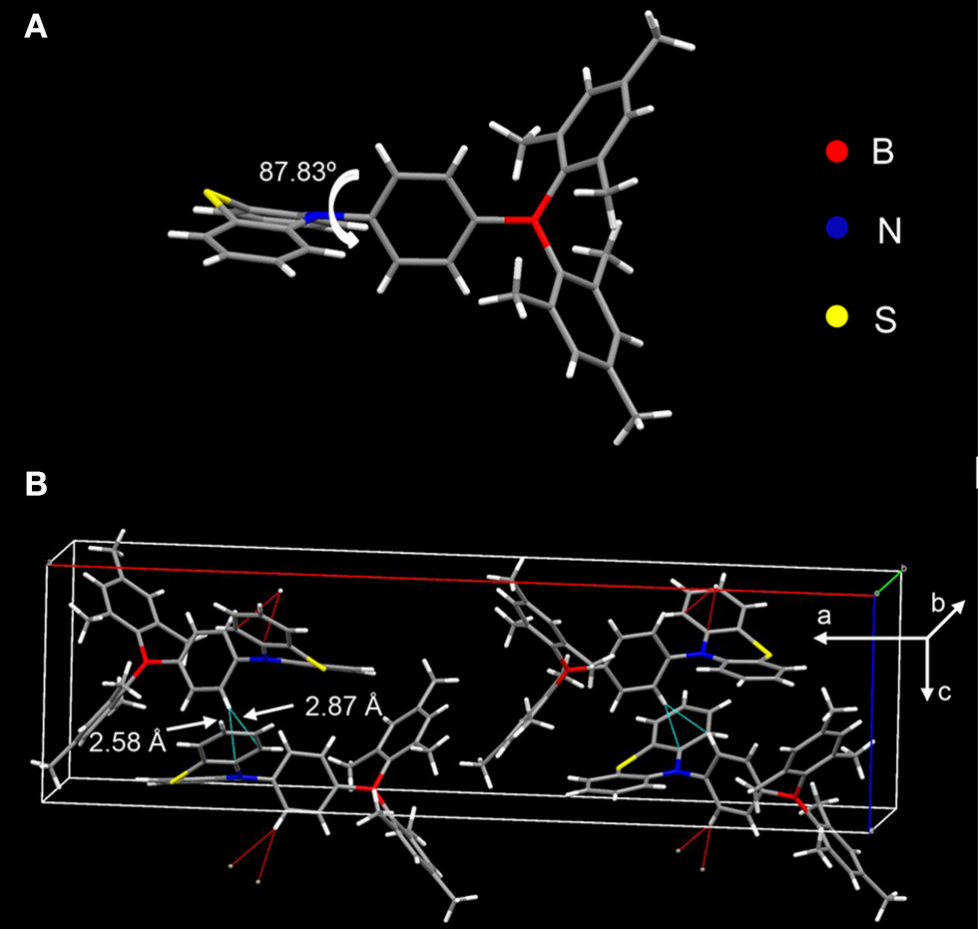
XRD of single crystal of PTZMes_2_B: **(A)** molecular conformation; **(B)** molecular packing in crystal.

The donor and acceptor are linked in a torsion fashion with a large dihedral angle of ~87.83° between them as evidenced by single crystal X-ray diffraction (XRD) shown in Figure 3A. As can be seen in Figure 3B, the adjacent molecules orientate inversely against each other. Considering the steric configuration of the acceptor Mes_2_B, a face-to-face packing between adjacent Mes_2_B would not be thermodynamically stable. Therefore, the acceptors avoid approaching each other and the arrangement of two adjacent molecules are in an inversion mode so that the resultant crystal can be in its thermodynamic stable state. In addition, PTZMes_2_B piles up without parallel face-to-face stacking. As a result, no π-π interactions or hydrogen bonds can be found in the crystal. Only CH–π interactions exist with a distance of ~2.58 and 2.87 Å as shown in Figure 3B. The weak intermolecular interactions mainly result from the bulky figure of the acceptor, which avoids close intermolecular contact. From the crystallography of PTZMes_2_B, we may further deduce that intermolecular interactions in thin film may also be weak, which is in favor of reducing both singlet and triplet quenching. Detailed information about the crystal can be seen in Table S4.

### Photophysical Properties

**Figure 4:**
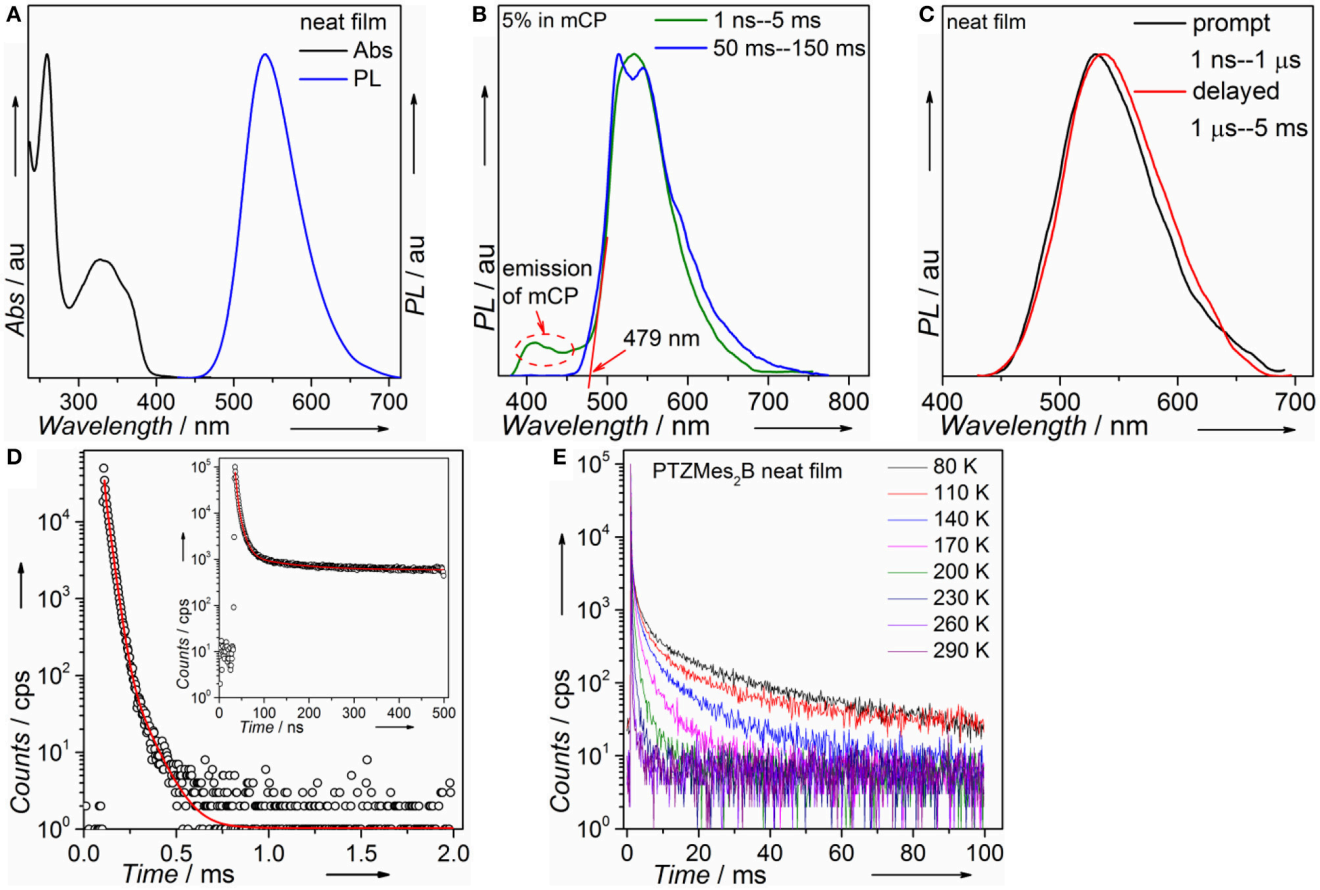
**(A)** Absorption and PL of PTZMes_2_B in neat film; **(B)** fluorescence (green line) and phosphorescence (blue line) of PTZMes_2_B doped film (wt. 5% in mCP); **(C)** prompt (dark line) and delayed (red line) fluorescence of PTZMes_2_B in neat film; **(D)** PL decay of PTZMes_2_B in neat film (inset: decay in the time range of 500 ns); **(E)** temperature-dependent transient PL spectra of PTZMes_2_B neat film under vacuum conditions.

Steady-state absorption and photoluminescence spectra of the PTZMes_2_B neat film recorded at ambient condition are shown in Figure 4A. The absorption band, showing vague vibronic structure peaking at ~360 and ~325 nm, can be assigned to the π → π^*^ transition of the molecule. The neat film of PTZMes_2_B shows intense green emission with an emission peak at ~540 nm and a relatively high PLQY of ~65%. The PLQY of doped films with various doping concentrations is also measured and displayed in Table S1 and Figure S3. The PLQY of doped films is higher than that of neat films. For example, the PLQY of wt. 10% (weight ratio) doped film is as high as 93%. Therefore, the neat film of PTZMes_2_B experiences a certain degree of concentration quenching. The decrease of PLQY of the neat film may be caused by concentration quenching of singlet or triplet excitons, or both. The deactivation of singlet excitons would lead to the quenching of both prompt and delayed fluorescence while the deactivation of triplet excitons would only lead to the quenching of delayed fluorescence. Thus, we measured transient PL spectra of these films at ambient condition. As shown in Figure S4, with increasing doping concentration, the lifetime of delayed component gradually shortened. This may be due to two possibilities. One is that the RISC of T_1_ → S_1_ is more efficient at aggregation state probably because either the Δ*E*_ST_ tends to be smaller or intermolecular spin-orbit coupling is stronger in aggregates. Thus, heavier doping films show shorter lifetime of the delayed component. The other possibility is that concentration caused triplet quenching that leads to a shorter lifetime of the delayed component at heavier doping level. Combined with the decreased PLQY of the neat film, we temporarily assume that the shortened lifetime of the delayed component is due to concentration quenching. More detailed experiments and analysis are ongoing to further investigate the influence of aggregates on the RISC of T_1_ → S_1_. Nevertheless, the neat film shows a relatively high PLQY of 65% and obvious delayed emission. If we consider exciton as hole–electron pair, ISC is essentially the process of spin flip of the excited electron. The binding energy between electron and hole of S_1_ state of common TADF emitters is weak as a result of almost completely separated hole–electron distributions (i.e., the CT attribute of S_1_ state). Hence, spin flip of the excited electron of S_1_ state occurs with high probability and therefore high efficiency of the ISC process. As a result, for TADF emitters, large amounts of S_1_ excitons would turn into T_1_ excitons *via* ISC. The PLQY of the TADF emitter would decrease dramatically if triplet exciton yields from S_1_ state are severely quenched and cannot be converted back to S_1_ state (Méhes et al., 2012). The relatively high PLQY of 65% and the obvious delayed component in the neat film show that large amounts of triplet excitons can still be converted back to S_1_ state instead of being quenched by intermolecular interactions such as dipole–dipole or π-π interactions. Though singlet and triplet quenching is inevitable in the neat film, our molecular design strategy, i.e., by introducing methyl groups to enhance steric repulsion among molecules, can help to alleviate concentration quenching in the neat film. To attain T_1_ energy as well as Δ*E*_ST_, time-resolved PL spectra of the doped film of PTZMes_2_B were measured at a low temperature of ~80 K, as can be seen in Figure 4B. The fluorescence or phosphorescence may be affected by aggregation mode. Therefore, we take the doped film of PTZMes_2_B as testing sample to try to eliminate the interference of aggregation. The doping concentration is wt. 5% and the host is mCP, which has higher triplet energy than PTZMes_2_B. The fluorescence and phosphorescence are collected in the time range of 1 ns−5 ms and 50–150 ms, respectively, when the excitation source is off. The phosphorescence of PTZMes_2_B shows a vibronic structure, indicating the LE character of T_1_ state. We find that the phosphorescence of PTZMes_2_B is similar to that of PTZP while different from that of Mes_2_BBr (Figure S5). Hence, we may conclude that the T_1_ state of PTZMes_2_B localizes on the donor moiety. NTOs calculations further confirm this speculation (Figure 2A). The first vibronic peak of PTZMes_2_B phosphorescence is ~515 nm, corresponding to an energy of 2.41 eV. The fluorescence spectra (1 ns−5 ms) are composed of combined emission of both mCP (fluorescence or phosphorescence or both) and PTZMes_2_B (prompt fluorescence or prompt + delayed fluorescence). The onset of the fluorescence is ~479 nm, and the energy is ~2.59 eV. Thus, the energy splitting Δ*E*_ST_ between S_1_ and T_1_ is determined to be 0.18 eV. Different experimental methods may produce different values of Δ*E*_ST_. The Δ*E*_ST_ herein derived from PL spectra of film can only be taken as one of the versions that may approach the true value of Δ*E*_ST_. Time-resolved PL spectra of the neat film of PTZMes_2_B recorded at ambient condition are displayed in Figure 4C. PTZMes_2_B exhibits delayed fluorescence (1 μs−5 ms) matching well with the prompt fluorescence (1 ns−1 μs), which is a persuasive evidence of TADF. Figure 4D is the PL decay of the PTZMes_2_B neat film measured at ambient condition. The inset is PL decay within 500 ns. The PL decay is far from finished in the time range of nanoseconds (Figure 4D, inset). The PL decay finishes in the timescale of milliseconds with a lifetime of 13.71, 77.45, and 891.40 μs. We have measured temperature-dependent steady-state PL spectra of doped and neat films from 80 to 290 K under vacuum conditions as shown in Figure S6. Generally, radiative rate constant, either fluorescence or phosphorescence, is independent of temperature. However, some non-radiative transitions are temperature dependent, such as thermal deactivations and RISC of T_1_ → S_1_, both of which are positively related to temperature. Therefore, for most TADF molecules, any PL intensity variations at different temperatures can be virtually ascribed to these two factors. However, thermal deactivations and RISC of T_1_ → S_1_ exert opposite effects on PL intensity as temperature changes. Figure S6a shows the temperature-dependent PL spectra of the doped film (wt. 10% in mCP, weight ratio). From 80 to 290 K, as the temperature increases, the emission intensity of mCP continues to decrease because thermal deactivations become more and more active, which would consume both singlets and triplets without producing photons and therefore weaken PL intensity. On the other hand, the situation is different for PTZMes_2_B. From 80 to 200 K, contrary to mCP, the PL intensity increases with temperature. This is because higher temperature can better activate RISC of T_1_ → S_1_ and the subsequent delayed fluorescence and therefore enhanced PL intensity. However, further temperature increase from 200 to 290 K results in the gradual decrease of PL intensity due to more and more active thermal deactivation. The neat film of PTZMes_2_B shows similar phenomenon as can be seen in Figure S6b: from 80 to 200 K, the PL intensity increases as a result of more and more efficient RISC process of T_1_ → S_1_. After 200 K, PL intensity gradually decreases due to thermal deactivation. We have also measured temperature-dependent transient PL spectra of the neat film under vacuum conditions as displayed in Figure 4E. At low temperature such as 80 K, PL decay is not completed within 100 ms and the long lifetime component beyond 100 ms can be attributed to phosphorescence, from which we can also conclude that the phosphorescence radiative rate must be very slow. In addition, even at temperature as low as 80 K, we can qualitatively conclude from Figure 4E that the proportion of the ultra-long lifetime component, i.e., the phosphorescence, is quite small, indicating that the phosphorescence process cannot compete with the RISC process of T_1_ → S_1_. Normally, the phosphorescence radiative rate constant is not affected by temperature. As temperature increases, thermal deactivations of triplet excitons as well as RISC of T_1_ → S_1_ become more and more active, and the slow phosphorescence process would not be able to compete with these processes. Indeed, with increasing temperature, the long lifetime component caused by phosphorescence gradually decreases and disappears at a temperature of 290 K. Actually, the PL decays completely within several milliseconds at 290 K. If phosphorescence exists at 290 K, considering its slow decay constant, the PL decay profile would not finish within several milliseconds. This means that the emission profile of Figure 4C does not contain any phosphorescence. In fact, under the same experimental conditions of Figure 4C at room temperature, no emission signal can be detected by delaying 50 ms. Thus, we can exclude the possibility of phosphorescence at room temperature and the long lifetime component of Figure 4D can be safely assigned to the TADF. Some basic photophysical data is listed in Table 1.

**Table 1:** Photophysical properties of PTZMes_2_B.

**Compound**	***T*_**d**_/*T*_**g**_/*T*_**c**_/*T*_m_^a^ [^**°**^C]**	**λ_max, abs_^b^ [nm]**	**λ_max, PL_^c^ [nm]**	**PLQY^d^ (%)**	**HOMO/LUMO^e^ [eV]**	**Δ*E*_**ST**_^f^ [eV]**
PTZMes_2_B	381/84/157/262	360/325	540	65	−5.03/−2.61	0.16^g^/0.18^h^

a *T_d_, decomposition temperature; T_g_, glass transition temperature; T_c_, crystalline temperature; T_m_, melting point*.

b *λ_max,abs_, absorption maximum in neat film*.

c *λ_max,PL_, emission peak in neat film*.

d *PLQY, photoluminescence quantum yield of neat film measured by integrating sphere*.

e *HOMO/LUMO energy levels estimated from cyclic voltammetry measurement*.

f *ΔE_ST_, energy splitting between S_1_ and T_1_ states*.

g *ΔE_ST_ derived from theoretical calculations*.

h *ΔE_ST_ calculated from emission difference between fluorescence and phosphorescence*.

### Electroluminescence Properties

**Figure 5:**
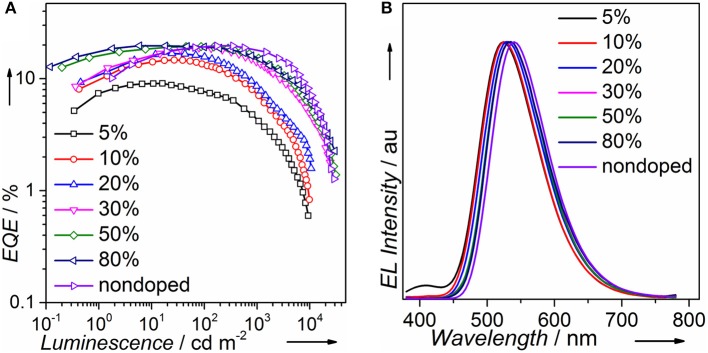
**(A)** EQE and **(B)** EL spectra at 7 V of PTZMes_2_B devices.

We fabricated both doped and non-doped EL devices *via* vacuum evaporation. The structure of doped devices is as follows: ITO/HATCN (6 nm)/TAPC (20 nm)/TCTA (10 nm)/mCP (10 nm)/mCP: wt. x% PTZMes_2_B (20 nm)/TPBi (40 nm)/LiF (1 nm)/Al (100 nm), where ITO (indium tin oxide) is the anode, TAPC [di-(4-(*N*,*N*-ditolyl-amino)-phenyl)cyclohexan] is the hole transporting layer (HTL), TCTA [tris(4-carbazoyl-9-ylphenyl)amine] is the buffer layer, mCP [1,3-bis(carbazol-9-yl)benzene] is the host material, TPBi [1,3,5-tris-(*N*-phenylbenzimidazol-2-yl)benzene] is the electron transporting layer (ETL), LiF is the electron injecting layer, and Al is the cathode (Figure S7). The emitting layer consists of mCP: wt. x% PTZMes_2_B with wt. x% indicating a doping ratio (wt.: weight ratio) of 5% (device A), 10% (device B), 20% (device C), 30% (device D), 50% (device E), and 80% (device F). The device performance can be seen in Figure S8. The turn-on voltage decreases as the doping concentration becomes higher as listed in Table 2. We believe that, at a low doping ratio such as wt. 5%, charges are injected directly into the host instead of the dopant. Excitons are formed on the host followed by energy transfer to the dopant. Accordingly, the turn-on voltage would be high because of the wide energy gap of the host. However, at high doping concentration, charges are mostly captured by the dopant and form excitons directly on dopant. Since the energy gap of dopant is much narrower than that of host, the turn-on voltage of heavily doped or non-doped devices is correspondingly lowered. Another interesting phenomenon is that the maximum EQE (EQE_max_) occurs at lower luminescence for low doping concentration devices such as wt. 5 and 10% but at higher luminescence for high doping concentration such as wt. 80% or non-doped devices as can be seen in Figure 5A. This essentially means that low doping concentration devices achieve EQE_max_ at low current density whereas high doping concentration or non-doped devices achieve EQE_max_ at high current density. Indeed, for example, EQE_max_ appears at *J* = 0.06 mA cm^−2^ for both wt. 5 and 10% devices and *J* = 0.27 mA cm^−2^ for the non-doped device. A similar phenomenon is also observed by Adachi et al. (Nakanotani et al., 2013). The host mCP does not contain any electron-withdrawing group that can lower its LUMO energy; thus electron injection from TPBi into mCP is not easy. However, the dopant shows both appropriate HOMO and LUMO energy levels that can facilitate balanced charge injection. Therefore, charge injection is more balanced for heavily doped or non-doped devices. Though we did not measure single carrier mobility of doped devices, it is reasonable to deduce that carrier transporting is also more balanced and the recombination zone is much broader, not only at the interface but also in the bulk of the emission layer, for heavily doped or non-doped devices according to the results of Adachi et al. (Nakanotani et al., 2013). Thus, higher current density would lead to unbalanced charge recombination and bring side effects such as charge-exciton quenching on device efficiency for low doping concentration devices. Moreover, the amount of dopant molecules in heavily doped or non-doped devices is much more than those in low doping concentration devices. If, ideally, each injected charge pair can form an exciton on molecules, then relatively low current density can already excite all dopant molecules when doping concentration is low. A higher current density would lead to redundant charges that would quench excitons. Hence, EQE_max_ occurs at lower current density for low doping concentration devices and higher current density for heavily or non-doped devices. The device performance enhances with increasing doping concentration (Figure 5A and Table 2). At low doping level, for example, wt. 5%, the corresponding EL spectra show a combined emission of both host and dopant with host emission being weaker than that of dopant (Figure 5B or Figure S8d). This means that (Adachi, 2014) excitons generated under electrical excitation are populated on both host and dopant in these doped devices (Reineke, 2014); singlet energy transfer from host to dopant is insufficient at low doping ratio. Generally, the effective radius of triplet energy transfer is much shorter than that of singlet energy transfer. The inadequate singlet energy transfer from host to dopant at a low doping ratio indicates that triplet energy transfer from host to dopant is even more insufficient. However, the host itself cannot utilize triplet excitons. As a result, triplet excitons localized on host are wasted, leading to incomplete exciton utilization and therefore relatively low efficiency. Indeed, the EQE is only ~9% for the wt. 5% doped device. At a higher doping ratio, wt. 10% for example, the host emission still exists, but is negligible, indicative of efficient singlet energy transfer from host to dopant, and the corresponding EQE is improved to ~14%. Further increasing doping ratio, i.e., wt. 20–80%, leads to the disappearance of host emission, which demonstrates more efficient singlet energy transfer from host to dopant. However, the EL originates from a singlet excited state. The triplet state is not emissive. Hence, the disappearance of host emission can only imply complete singlet energy transfer. As stated above, the effective radius of triplet energy transfer is much shorter than that of singlet energy transfer. It would be arbitrary to deduce that triplet excitons formed on host are 100% transferred to the dopant from the phenomenon of the disappearance of host emission in the EL spectra. Accordingly, for doping ratio ranging from wt. 20 to 80%, though the host emission vanishes, the EQE continues to improve, in part owing to enhanced triplet energy transfer from host to dopant as the doping ratio increases. At a doping ratio as high as wt. 80%, the EQE can reach ~19.73%. We also fabricated doped devices based on other hosts such as DPEPO and mCBP and observed similar phenomenon as presented in Figures S9, S10 and Tables S2, S3. The fact that a heavily doped device can achieve better device performance promotes us to explore the potential application of PTZMes_2_B in the non-doped device. The device structure of the non-doped device is as follows: ITO/HATCN (6 nm)/TAPC (25 nm)/TCTA (15 nm)/PTZMes_2_B (20 nm)/TPBi (40 nm)/LiF (1 nm)/Al (100 nm). The turn-on voltage is 2.8 V and the maximum luminescence is ~28,000 cd m^−2^. The non-doped device shows green emission with EL peak of 540 nm and CIE coordinates of (0.37, 0.57). The maximum current efficiency (CE) and EQE are 62.88 cd A^−1^ and 19.66%, respectively, which are obtained at a luminescence of ~170 cd m^−2^. At a high luminescence of ~1,500 cd m^−2^, the EQE is still as high as 17.31%, showing an efficiency roll-off of about 12% compared with the maximum EQE of 19.66%. The discussion of both doped and non-doped devices is not intended to highlight that the performance of non-doped device is superior over doped devices but to emphasize the feasibility of PTZMes_2_B as a non-doped emitter. This result verifies that our molecular design strategy works. The methyl in Mes_2_B can function as an intermolecular steric group that may relieve triplet exciton quenching caused by intermolecular interactions. However, severe efficiency roll-off still exists when luminescence is much higher, for example, after 5,000 cd m^−2^. This may be caused by the slow RISC of T_1_ → S_1_, which is an intrinsic defect of TADF emitter. Large amounts of triplet excitons would accumulate at high current density due to their long-lived attribute, resulting in various active triplet quenching processes such as triplet–triplet annihilation, triplet-charge quenching, etc., and therefore serious efficiency roll-off. The fundamental solution toward relieving efficiency roll-off at high luminescence is by accelerating the T_1_ → S_1_ RISC process to the extent of several nanoseconds so that triplets can transform to singlets in time instead of being quenched. This is a great challenge beyond this paper. Nevertheless, high EQE and relatively small efficiency roll-off are realized in the non-doped device, which, from our point of view, is a tiny advancement among TADF materials.

**Table 2:** EL performance of PTZMes_2_B-based devices.

**Device**	***V*_on_^a^ [V]**	***L*_max_^b^ [cd m^**−2**^]**	**CE_max_^c^ [cd A^**−1**^]**	**EQE^d^ [%]**	**EL λ_max_^e^ [nm]**	**CIE^f^ [*x*, *y*]**
				**max/100/1000**		
wt. 5%	4.4	9,388	26.44	9.07/7.78/4.18	525	0.31, 0.54
wt. 10%	4.2	9,831	45.05	14.66/13.52/7.06	525	0.31, 0.55
wt. 20%	4.0	10,589	52.65	16.79/15.65/8.60	530	0.33, 0.55
wt. 30%	3.6	24,467	61.09	18.90/18.71/13.52	535	0.35, 0.56
wt. 50%	3.6	31,006	62.47	19.40/19.20/15.22	535	0.35, 0.56
wt. 80%	3.4	29,920	63.06	19.73/19.22/14.91	536	0.36, 0.56
Non-doped	2.8	28,845	62.88	19.66/19.66/17.31	540	0.37, 0.57

a V_on_, turn-on voltage at the luminescence of ~1 cd m^-2.^

b *L_max_, maximum luminescence*.

c *CE_max_, maximum current efficiency*.

d EQE max/1,000/10,000, EQE of maximum/at 1,000 cd m^-2^/10,000 cd m^-2.^

e *EL λ_max_, emission peak of EL spectrum at 7 V*.

f *CIE coordinates at 7 V*.

## Conclusion

In conclusion, we have demonstrated a phenothiazinen-dimesitylarylborane (PTZMes_2_B)-based twisted D-A molecule with TADF for high-performance non-doped OLEDs with reduced efficiency roll-off. PTZMes_2_B shows a high PLQY of 65% in the neat film. A maximum EQE of 19.66% is obtained at a luminescence of 170 cd m^−2^ in the non-doped device. A relatively small efficiency roll-off is achieved. The EQE is still as high as 17.31% at a high luminescence of 1,500 cd m^−2^. Our work proves that introducing steric groups to reduce intermolecular interactions is in favor of acquiring a high PLQY in the neat film as well as alleviating triplet exciton quenching at high current density, both of which are crucial in terms of improving device efficiency and reducing efficiency roll-off. However, a fundamental solution to reduce efficiency roll-off of TADF materials rests upon accelerating the RISC process of T_1_ → S_1_ to the timescale of several nanoseconds, which remains a great challenge for future material development.

## Author Contributions

XT designed and synthesized the TADF compound and carried out photophysical characterization and wrote the manuscript. YT helped in material synthesis and structural characterization. FL helped in material synthesis and purification. HL helped in device fabrication and characterization. JL helped in CV measurement. QP helped in photophysical characterization. XH helped in theoretical calculations. PL helped revise the manuscript and gave many suggestions.

### Conflict of Interest Statement

The authors declare that the research was conducted in the absence of any commercial or financial relationships that could be construed as a potential conflict of interest.

## References

[B1] Adachi C. (2014). Third-generation organic electroluminescence materials. Jpn. J. Appl. Phys. 53:060101 10.7567/JJAP.53.060101

[B2] Bonn A. G. Wenger O. S. (2015). Charge transfer emission in oligotriarylamine–triarylborane compounds. J. Org. Chem. 80, 4097–4107. 10.1021/acs.joc.5b00416 25843000

[B3] Chen D.-G. Lin T.-C. Chen C.-L. Chen Y.-T. Chen Y.-A. Lee G.-H.. (2018). Optically triggered planarization of boryl-substituted phenoxazine: another horizon of TADF molecules and high-performance OLEDs. ACS Appl. Mater. Interfaces 10, 12886–12896. 10.1021/acsami.8b00053 29582654

[B4] Chen H.-W. Lee J.-H. Lin B.-Y. Chen S. Wu S.-T. (2018). Liquid crystal display and organic light-emitting diode display: present status and future perspectives. Light Sci. Appl. 7:17168. 10.1038/lsa.2017.168 30839536 PMC6060049

[B5] Chen X.-L. Jia J.-H. Yu R. M. Liao J.-Z. Yang M.-X. Lu C.-Z. (2017). Combining charge-transfer pathways to achieve unique thermally activated delayed fluorescence emitters for high-performance solution-processed, non-doped blue OLEDs. Angew. Chem. Int. Ed. 56, 15006–15009. 10.1002/anie.201709125 28990260

[B6] D'Aléo A. Sazzad M. H. Kim D. H. Choi E. Y. Wu J. W. Canard G.. (2017). Boron difluoride hemicurcuminoid as an efficient far red to near-infrared emitter: toward OLEDs and laser dyes. Chem. Commun. 53, 7003–7006. 10.1039/c7cc01786c 28513655

[B7] Dou C. D. Long X. J. Ding Z. C. Xie Z. Y. Liu J. Wang L. X. (2016). An electron-deficient building block based on the B?N unit: an electron acceptor for all-polymer solar cells. Angew. Chem. Int. Ed. 55, 1436–1440. 10.1002/anie.201508482 26663513

[B8] Endo A. Ogasawara M. Takahashi A. Yokoyama D. Kato Y. Adachi C. (2009). Thermally activated delayed fluorescence from Sn^4+^-porphyrin complexes and their application to organic light-emitting diodes—a novel mechanism for electroluminescence. Adv. Mater. 21, 4802–4806. 10.1002/adma.200900983 21049498

[B9] Endo A. Sato K. Yoshimura K. Kai T. Kawada A. Miyazaki H. (2011). Efficient up-conversion of triplet excitons into a singlet state and its application for organic light emitting diodes. Appl. Phys. Lett. 98:083302 10.1063/1.3558906

[B10] Guo F. Karl A. Xue Q.-F. Tam K. C. Forberich K. Brabec C. J. (2017a). The fabrication of color-tunable organic light-emitting diode displays *via* solution processing. Light Sci. Appl. 6:e17094. 10.1038/lsa.2017.94 30167215 PMC6062041

[B11] Guo J. Li X.-L. Nie H. Luo W. Gan S. Hu S. (2017b). Achieving high-performance nondoped OLEDs with extremely small efficiency roll-off by combining aggregation-induced emission and thermally activated delayed fluorescence. Adv. Funct. Mater. 27:1606458 10.1002/adfm.201606458

[B12] Guo J. Li X.-L. Nie H. Luo W. Hu R. Qin A. (2017c). Robust luminescent materials with prominent aggregation-induced emission and thermally activated delayed fluorescence for high performance organic light-emitting diodes. Chem. Mater. 29, 3623–3631. 10.1021/acs.chemmater.7b00450

[B13] Hatakeyama T. Shiren K. Nakajima K. Nomura S. Nakatsuka S. Kinoshita K.. (2016). Ultrapure blue thermally activated delayed fluorescence molecules: Efficient HOMO–LUMO separation by the multiple resonance effect. Adv. Mater. 28, 2777–2781. 10.1002/adma.201505491 26865384

[B14] Hirai H. Nakajima K. Nakatsuka S. Shiren K. Ni J. P. Nomura S.. (2015). One-step borylation of 1,3-diaryloxybenzenes towards efficient materials for organic light-emitting diodes. Angew. Chem. Int. Ed. 54, 13581–13585. 10.1002/anie.201506335 26380959

[B15] Jia W. L. Feng X. D. Bai D. R. Lu Z. H. Wang S. N. Vamvounis G. (2005). Mes_2_B(*p-*4,4-biphenyl-NPh(1-naphthyl)): a multifunctional molecule for electroluminescent devices. Chem. Mater. 17, 164–170. 10.1021/cm048617t

[B16] Kim D.-H. D'Aléo A. Chen X.-K. Sandanayaka A. D. S. Yao D. D. Zhao L. (2018). High-efficiency electroluminescence and amplified spontaneous emission from a thermally activated delayed fluorescent near-infrared emitter. Nat. Photon. 12, 98–104. 10.1038/s41566-017-0087-y

[B17] Kitamoto Y. Namikawa T. Ikemizu D. Miyata Y. Suzuki T. Kita H. (2015). Light blue and green thermally activated delayed fluorescence from 10H-phenoxaborin-derivatives and their application to organic light-emitting diodes. J. Mater. Chem. C 3, 9122–9130. 10.1039/c5tc01380a

[B18] Kitamoto Y. Namikawa T. Suzuki T. Miyata Y. Kita H. Sato T. (2016a). Design and synthesis of efficient blue thermally activated delayed fluorescence molecules bearing triarylborane and 10,10-dimethyl-5,10-dihydrophenazasiline moieties. Tetrahedron Lett. 57, 4914–4917. 10.1016/j.tetlet.2016.09.072

[B19] Kitamoto Y. Namikawa T. Suzuki T. Miyata Y. Kita H. Sato T. (2016b). Dimesitylarylborane-based luminescent emitters exhibiting highlyefficient thermally activated delayed fluorescence for organic light emitting diodes. Org. Electron. 34, 208–217. 10.1016/j.orgel.2016.04.030

[B20] Komino T. Nomura H. Koyanagi T. Adachi C. (2013). Suppression of efficiency roll-off characteristics in thermally activated delayed fluorescence based organic light-emitting diodes using randomly oriented host molecules. Chem. Mater. 25, 3038–3047. 10.1021/cm4011597

[B21] Lee S. Y. Yasuda T. Nomura H. Adachi C. (2012). High-efficiency organic light-emitting diodes utilizing thermally activated delayed fluorescence from triazine-based donor–acceptor hybrid molecules. Appl. Phys. Lett. 101:093306 10.1063/1.4749285

[B22] Lee Y. H. Park S. Oh J. Shin J. W. Jung J. Yoo S.. (2017). Rigidity-induced delayed fluorescence by ortho donor-appended triarylboron compounds: record-high efficiency in pure blue fluorescent organic light-emitting diodes. ACS Appl. Mater. Interfaces 9, 24035–24042. 10.1021/acsami.7b05615 28653832

[B23] Li J. Ding D. Tao Y. Wei Y. Chen R. Xie L.. (2016). A significantly twisted spirocyclic phosphine oxide as a universal host for high-efficiency full-color thermally activated delayed fluorescence diodes. Adv. Mater. 28, 3122–3130. 10.1002/adma.201506286 26923460

[B24] Lien Y. J. Lin T.-C. Yang C.-C. Chiang Y.-C. Chang C.-H. Liu S.-H.. (2017). First N-borylated emitters displaying highly efficient thermally activated delayed fluorescence and high-performance OLEDs. ACS Appl. Mater. Interfaces 9, 27090–27101. 10.1021/acsami.7b08258 28731681

[B25] Liu B.-Q. Wang L. Gao D.-Y. Zou J.-H. Ning H.-L. Peng J.-B.. (2016). Extremely high-efficiency and ultrasimplified hybrid white organic light-emitting diodes exploiting double multifunctional blue emitting layers. Light Sci. Appl. 5:e16137. 10.1038/lsa.2016.137 30167184 PMC6059940

[B26] Matsuo K. Yasuda T. (2017). Enhancing thermally activated delayed fluorescence characteristics by intramolecular B–N coordination in a phenylpyridine-containing donor–acceptor π-system. Chem. Commun. 53, 8723–8726. 10.1039/c7cc04875k 28726860

[B27] Méhes G. Nomura H. Zhang Q. Nakagawa T. Adachi C. (2012). Enhanced electroluminescence efficiency in a spiro-acridine derivative through thermally activated delayed fluorescence. Angew. Chem. Int. Ed. 51, 11311–11315. 10.1002/anie.201206289 23042555

[B28] Miao Y. Wang K. Gao L. Zhao B. Wang H. Zhu F. (2018a). Precise manipulation of the carrier recombination zone: a universal novel device structure for highly efficient monochrome and white phosphorescent organic light-emitting diodes with extremely small efficiency roll-off. J. Mater. Chem. C 6, 8122–8134. 10.1039/C8TC02479K

[B29] Miao Y. Wang K. Zhao B. Gao L. Tao P. Liu X. (2018b). High-efficiency/CRI/color stability warm white organic light-emitting diodes by incorporating ultrathin phosphorescence layers in a blue fluorescence layer. Nanophotonics 7, 295–304. 10.1515/nanoph-2017-0021

[B30] Na Y.-J. Song W. Lee J. Y. Hwang S.-H. (2015). Synthesis of dibenzothiophene-based host materials with a dimesitylborane substituent and their green PHOLED performances. Dalton Trans. 44, 8360–8363. 10.1039/c4dt03700f 25757853

[B31] Nakanotani H. Furukawa T. Hosokai T. Hatakeyama T. Adachi C. (2017). Light amplification in molecules exhibiting thermally activated delayed fluorescence. Adv. Optical Mater. 5:1700051 10.1002/adom.201700051

[B32] Nakanotani H. Masui K. Nishide J. Shibata T. Adachi C. (2013). Promising operational stability of high-efficiency organic light-emitting diodes based on thermally activated delayed fluorescence. Sci. Rep. 3:2127. 10.1038/srep02127 23820465 PMC3705585

[B33] Neena K. K. Pagidi S. Dipak K. Thilagar P. (2017). Diarylboryl-phenothiazine based multifunctional molecular siblings. Chem. Commun. 53, 3641–3644. 10.1039/C6CC09717K 28058430

[B34] Numata M. Yasudaab T. Adachi C. (2015). High efficiency pure blue thermally activated delayed fluorescence molecules having 10H-phenoxaborin and acridan units. Chem. Commun. 51, 9443–9446. 10.1039/c5cc00307e 25959457

[B35] Park I. S. Numata M. Adachi C. Yasuda T. (2016). A phenazaborin-based high efficiency blue delayed fluorescence material. Bull. Chem. Soc. Jpn. 89, 375–377. 10.1246/bcsj.20150399

[B36] Pomrnerehne J. Vestweber H. Gun W. Muhrt R. F. Bässler H. Porsch M. (1995). Efficient two layer LEDs on a polymer blend basis. Adv. Mater. 7, 551–554. 10.1002/adma.19950070608

[B37] Rao J. Zhao C. Wang Y. Bai K. Wang S. Ding J. (2019). Achieving deep-blue thermally activated delayed fluorescence in nondoped organic light-emitting diodes through a spiro-blocking strategy. ACS Omega 4, 1861–1867. 10.1021/acsomega.8b03296 PMC6648766 31459441

[B38] Reineke S. (2014). Phosphorescence meets its match. Nat. Photon. 8, 269–270. 10.1038/nphoton.2014.78

[B39] Shirota Y. Kinoshita M. Noda T. Okumoto K. Ohara T. (2000). A novel class of emitting amorphous molecular materials as bipolar radical formants: 2-4-[bis(4-methylphenyl)amino]phenyl-5-(dimesitylboryl)thiophene and 2-4-[bis(9,9-dimethylfluorenyl)amino]phenyl-5-(dimesitylboryl)thiophene. J. Am. Chem. Soc. 122, 11021–11022. 10.1021/ja0023332

[B40] Shiu Y.-J. Cheng Y.-C. Tsai W.-L. Wu C.-C. Chao C.-T. Lu C. W.. (2016). Pyridyl pyrrolide boron complexes: the facile generation of thermally activated delayed fluorescence and preparation of organic light-emitting diodes. Angew. Chem. Int. Ed. 55, 3017–3021. 10.1002/anie.201509231 26822378

[B41] Suzuki K. Kubo S. Shizu K. Fukushima T. Wakamiya A. Murata Y.. (2015). Triarylboron-based fluorescent organic light-emitting diodes with external quantum efficiencies exceeding 20%. Angew. Chem. Int. Ed. 54, 15231–15235. 10.1002/anie.201508270 26563845

[B42] Uoyama H. Goushi K. Shizu K. Nomura H. Adachi A. (2012). Highly efficient organic light-emitting diodes from delayed fluorescence. Nature 492, 234–238. 10.1038/nature11687 23235877

[B43] Welch G. C. Juan R. R. S. Masuda J. D. Stephan D. W. (2006). Reversible, metal-free hydrogen activation. Science 314, 1124–1126. 10.1126/science.1134230 17110572

[B44] Yamaguchi S. Akiyama S. Tamao K. (2001). Colorimetric fluoride ion sensing by boron-containing π-electron system. J. Am. Chem. Soc. 123, 11372–11375. 10.1021/ja015957w 11707112

[B45] Yang Y. Zhao L. Wang S. Ding J. Wang L. (2018). Red-emitting thermally activated delayed fluorescence polymers with poly(fluorene-*co*-3,3′-dimethyl diphenyl ether) as the backbone. Macromolecules 51, 9933–9942. 10.1021/acs.macromol.8b02050

[B46] Yao L. Xue S. Wang Q. Dong W. Yang W. Wu H.. (2012). RGB small molecules based on a bipolar molecular design for highly efficient solution-processed single-layer OLEDs. Chem. Eur. J. 18, 2707–2714. 10.1002/chem.201101476 22282319

[B47] Zhang D. Cai M. Zhang Y. Bin Z. Zhang D. Duan L. (2016). Simultaneous enhancement of efficiency and stability of phosphorescent OLEDs based on efficient Förster energy transfer from interface exciplex. ACS Appl. Mater. Interfaces 6, 3825–3832. 10.1021/acsami.5b10561 26800082

[B48] Zhang D. Duan L. Zhang Y. Cai M. Zhang D. Qiu Y. (2015). Highly efficient hybrid warm white organic light-emitting diodes using a blue thermally activated delayed fluorescence emitter: exploiting the external heavy-atom effect. Light Sci. Appl. 4:e232 10.1038/lsa.2015.5

[B49] Zhang J. Ding D. Wei Y. Han F. Xu H. Huang W. (2016). Multiphosphine-oxide hosts for ultralow-voltage-driven true-blue thermally activated delayed fluorescence diodes with external quantum efficiency beyond 20%. Adv. Mater. 28, 479–485. 10.1002/adma.201502772 26588189

[B50] Zhang Q. Kuwabara H. Potscavage W. J. Huang S. Hatae Y. Shibata T.. (2014a). Anthraquinone-based intramolecular charge-transfer compounds: computational molecular design, thermally activated delayed fluorescence, and highly efficient red electroluminescence. J. Am. Chem. Soc. 136, 18070–11808. 10.1021/ja510144h 25469624

[B51] Zhang Q. Li B. Huang S. Nomura H. Tanaka H. Adachi C. (2014b). Efficient blue organic light-emitting diodes employing thermally activated delayed fluorescence. Nat. Photon. 8, 326–332. 10.1038/nphoton.2014.12

[B52] Zhang Q. Li J. Shizu K. Huang S. Hirata S. Miyazaki H.. (2012). Design of efficient thermally activated delayed fluorescence materials for pure blue organic light emitting diodes. J. Am. Chem. Soc. 134, 14706–14709. 10.1021/ja306538w 22931361

