# Supplementary material for: Phenothiazinen-Dimesitylarylborane-Based Thermally Activated Delayed Fluorescence: High-Performance Non-doped OLEDs With Reduced Efficiency Roll-Off at High Luminescence
Source: Front Chem. 2019 May 29;7:373. doi: 10.3389/fchem.2019.00373 (PMC6548864; doi:10.3389/fchem.2019.00373)
Supplement: Supplementary file 1 [file Data_Sheet_1.docx]

Supplementary Material

Phenothiazinen-Dimesitylarylborane based Thermally Activated Delayed Fluorescence: High Performance Nondoped OLEDs with Reduced Efficiency Roll-Off at High Luminescence

Xiangyang Tang^1#^, Yanchun Tao^1#^, Hui Liu^1^, Futong Liu^1^, Xin He^1^, Qiming Peng^2^, Jinyu Li^1^, Ping Lu^1*^

*** Correspondence:** Ping Lu: [lup@jlu.edu.cn](mailto:lup@jlu.edu.cn)


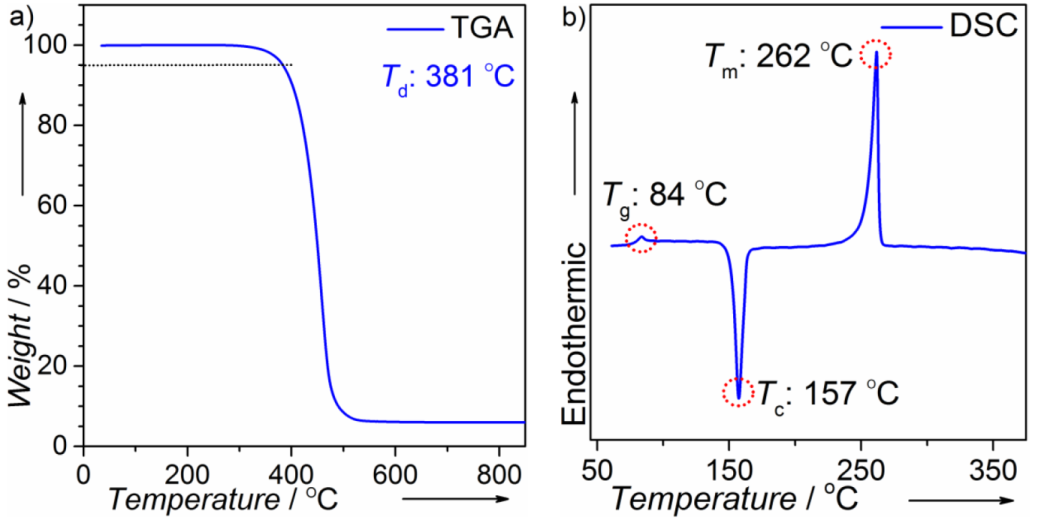


**Figure S1** (a) TGA and (b) DSC of PTZMes_2_B.


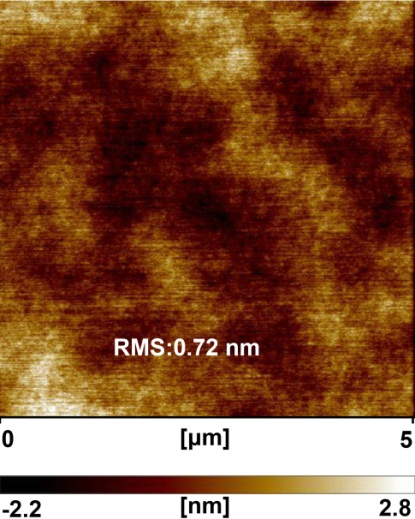


**Figure S2.** AFM image (5μm × 5 μm) of PTZMes_2_B.

**Table S1**. PLQY of doped films.

| Doping concentration  (weight ratio) | 5% | 10% | 20% | 30% | 50% | 80% | 100% |
| --- | --- | --- | --- | --- | --- | --- | --- |
| PLQY (%) | 86 | 93 | 91 | 89 | 78 | 77 | 65 |





**Figure S3.** PLQY of doped films.





**Figure S4.** Transient PL spectra of doped and neat films.





**Figure S5.** Phosphorescence of Mes_2_BBr and PTZP.


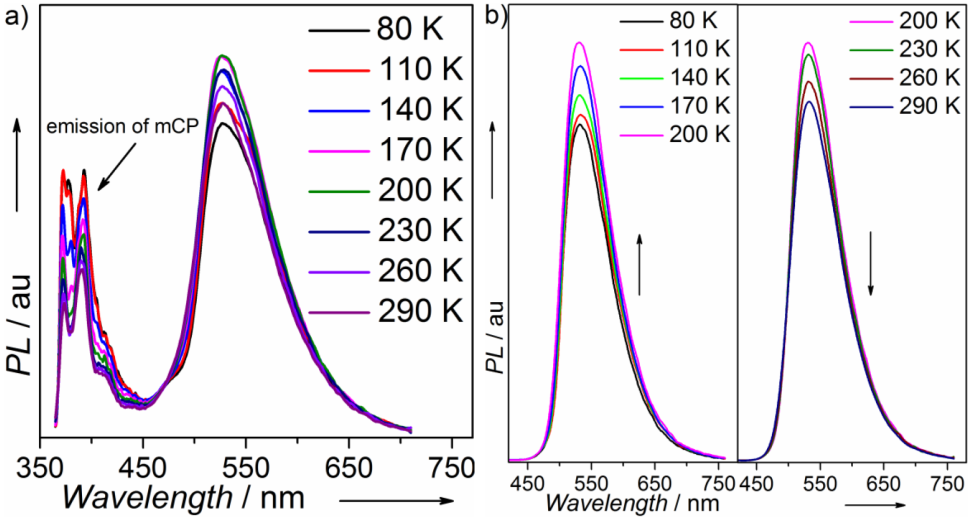


**Figure S6.** Temperature-dependent steady state PL spectra of (a) wt. 10% doped and (b) neat films from 80 K to 290 K under vacuum condition.


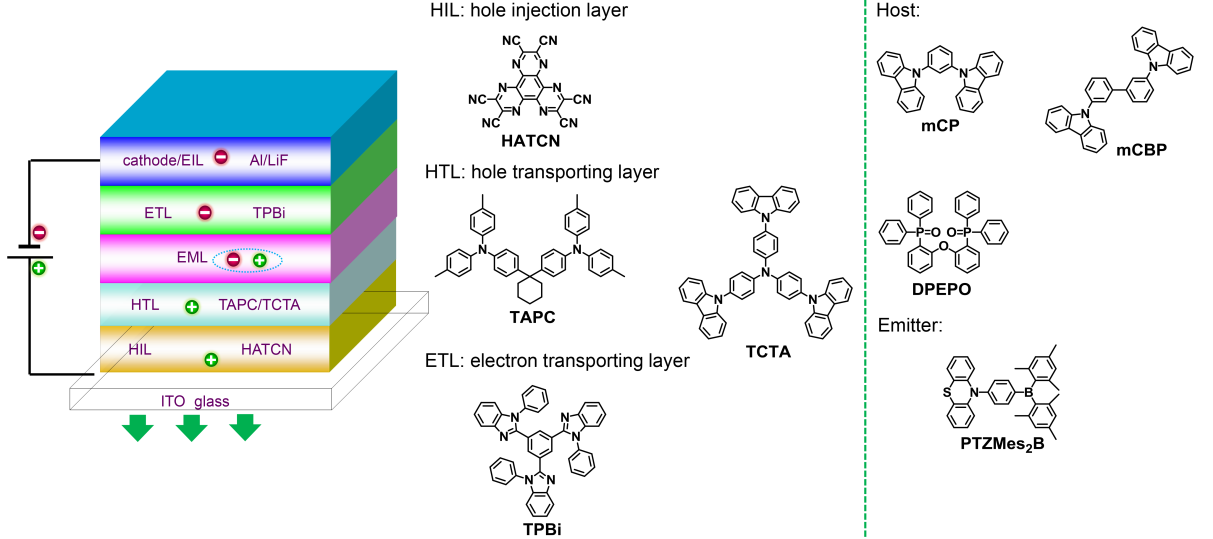


**Figure S7.** Device configuration and chemical structure.




























**Figure S8.** (a) *I-V-L* (b) luminescence efficiency and (c) power efficiency of PTZMes_2_B devices; EL spectra of (d) wt. 5% (e) wt. 10% (f) wt. 20% (g) wt. 30% (h) wt. 50% (i) wt. 80% and (j) nondoped devices.


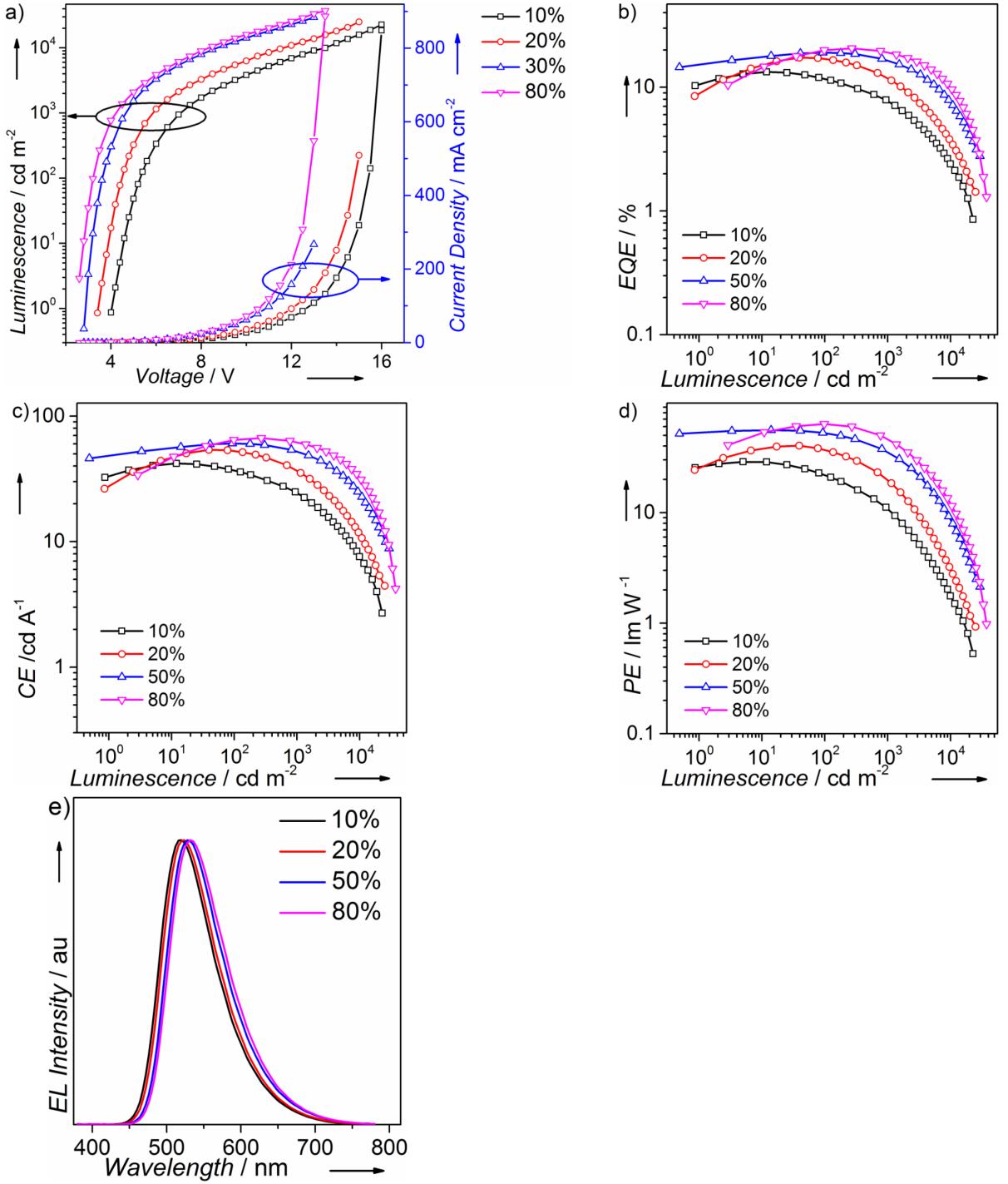


**Figure S9.** Device performance of PTZMes_2_B-mCBP doped devices: (a) *I-V-L*, (b) EQE-Luminescence, (c) current efficiency-luminescence, (d) power efficiency-luminescence and (e) PL spectra at driving voltage of 7 V.


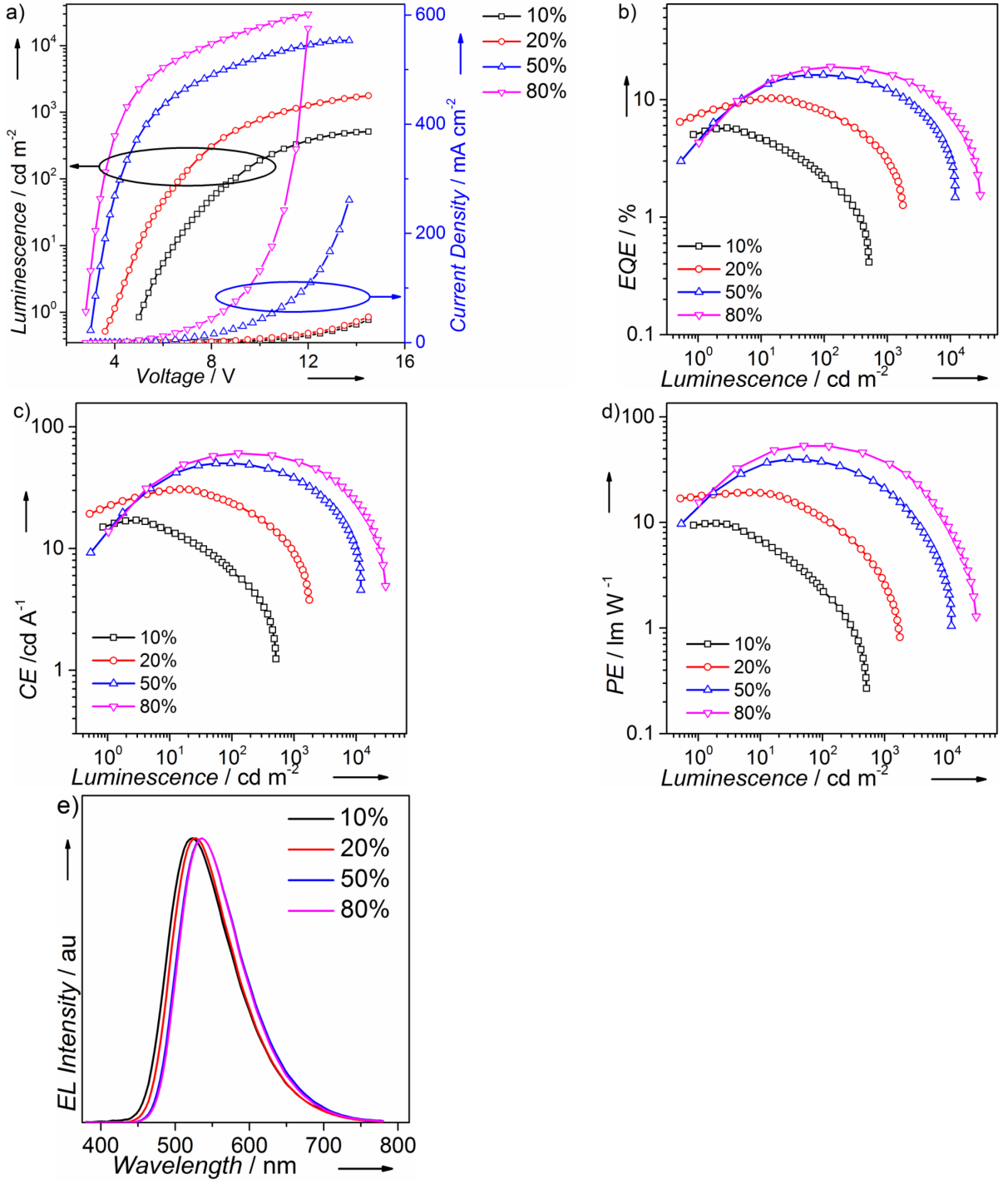


**Figure S10.** Device performance of PTZMes_2_B-DPEPO doped devices: (a) *I-V-L*, (b) EQE-Luminescence, (c) current efficiency-luminescence, (d) power efficiency-luminescence and (e) PL spectra at driving voltage of 7 V.

**Table S2.** EL performance of PTZMes_2_B-mCBP based doped devices.

| Device | *V*_on_^a)^  [V] | *L*_max_^b)^  [cd m^-2^] | CE_max_^c)^  [cd A^-1^] | EQE^d)^ [%] | EL *λ*_max_^e)^  [nm] | CIE^f)^  [x, y] |
| --- | --- | --- | --- | --- | --- | --- |
|  |  |  |  | max/100/1000 |  |  |
| wt. 10% | 4.0 | 22847 | 41.96 | 13.30/11.35/7.88 | 518 | 0.32, 0.58 |
| wt. 20% | 3.4 | 25094 | 53.87 | 17.33/16.55/11.35 | 522 | 0.32, 0.58 |
| wt. 50% | 3.0 | 29459 | 60.41 | 19.03/19.03/16.98 | 529 | 0.34, 0.58 |
| wt. 80% | 2.8 | 37378 | 66.81 | 20.63/19.91/18.36 | 533 | 0.36, 0.58 |

**Table S3.** EL performance of PTZMes_2_B-DPEPO based doped devices.

| Device | *V*_on_^a)^  [V] | *L*_max_^b)^  [cd m^-2^] | CE_max_^c)^  [cd A^-1^] | EQE^d)^ [%] | EL *λ*_max_^e)^  [nm] | CIE^f)^  [x, y] |
| --- | --- | --- | --- | --- | --- | --- |
|  |  |  |  | max/1000/10000 |  |  |
| wt. 10% | 5.0 | 512 | 17.10 | 5.73/2.11/- | 522 | 0.32, 0.54 |
| wt. 20% | 3.8 | 1771 | 30.72 | 10.29/7.77/2.99 | 526 | 0.34, 0.56 |
| wt. 50% | 3.2 | 11910 | 50.26 | 16.27/16.27/12.33 | 534 | 0.37, 0.57 |
| wt. 80% | 2.8 | 29803 | 60.62 | 18.99/18.99/16.13 | 534 | 0.37, 0.57 |

Abbreviations: ^a)^*V*_on_: turn-on voltage at the luminescence of ~ 1 cd m^-2^; ^b)^*L*_max_: maximum luminescence; ^c)^CE_max_: maximum current efficiency; ^d)^EQE max/1000/10000: EQE of maximum / at 1000 cd m^-2^ / 10000 cd m^-2^; ^e)^EL *λ*_max_: emission peak of EL spectrum at 7 V; ^f)^CIE coordinates at 7 V.

**Table S4.** Crystal data and structure refinement for PTZMes_2_B.

| Empirical formula | C_36_H_34_BNS |
| --- | --- |
| Formula weight | 523.51 |
| Temperature | 293(2) K |
| Wavelength | 0.71073 A |
| Crystal system, space group | Orthorhombic, Pca2(1) |
| Unit cell dimensions | a = 34.6477(16) A α = 90°  b = 8.5153(4) A β = 90°  c = 9.9114(4) A γ = 90° |
| Volume | 2924.2(2) A^3^ |
| Z, Calculated density | 4, 1.189 mg m^-3^ |
| Absorption coefficient | 0.136 mm^-1^ |
| F(000) | 1112 |
| Crystal size | 0.130 x 0.120 x 0.100 mm |
| θ range for data collection | 2.972°-28.276° |
| Limiting indices | -46<=h<=46, -11<=k<=11, -13<=l<=9 |
| Final R indices [I>2σ(I)] | R1 = 0.0483, wR2 = 0.1266 |
| R indices (all data) | R1 = 0.0598, wR2 = 0.1355 |
